# Subzero project: comparing trace element profiles of enriched mitochondria fractions from frozen and fresh liver tissue

**DOI:** 10.1007/s00216-024-05400-y

**Published:** 2024-07-03

**Authors:** Tom Heinze, Franziska Ebert, Christiane Ott, Judith Nagel, Carola Eberhagen, Hans Zischka, Tanja Schwerdtle

**Affiliations:** 1 Department of Food Chemistry, Institute of Nutritional Science, University of Potsdam, Nuthetal, Germany; 2 TraceAge – DFG Research Unit on Interactions of Essential Trace Elements in Healthy and Diseased Elderly (FOR 2558), Berlin-Potsdam-Jena-Wuppertal, Germany; 3 Department of Molecular Toxicology, German Institute of Human Nutrition, Nuthetal, Germany; 4 Institute of Toxicology and Environmental Hygiene, Technical University Munich, School of Medicine and Health, Munich, Germany; 5 Institute of Molecular Toxicology and Pharmacology, Helmholtz Munich, Munich, Germany; 6 German Federal Institute for Risk Assessment (BfR), Berlin, Germany

**Keywords:** Mitochondria isolation, Trace elements, Method development, Manganese, Iron, Copper

## Abstract

From organs to subcellular organelles, trace element (TE) homeostasis is fundamental for many physiological processes. While often overlooked in early stages, manifested TE disbalance can have severe health consequences, particularly in the context of aging or pathological conditions. Monitoring TE concentrations at the mitochondrial level could identify organelle-specific imbalances, contributing to targeted diagnostics and a healthier aging process. However, mitochondria isolation from frozen tissue is challenging, as it poses the risk of TE losses from the organelles due to cryodamage, but would significantly ease routine laboratory work. To address this, a novel method to isolate an enriched mitochondria fraction (EMF) from frozen tissue was adapted from already established protocols. Validation of manganese (Mn), iron (Fe), and copper (Cu) quantification via inductively coupled plasma tandem mass spectrometry (ICP-MS/MS) showed sufficiently low quantification limits for EMF TE analysis. Successful mitochondrial enrichment from frozen liver samples was confirmed via immunoblots and transmission electron microscopy (TEM) revealed sufficient structural integrity of the EMFs. No significant differences in EMF TEs between frozen and fresh tissue were evident for Mn and Cu and only slight decreases in EMF Fe. Consequently, EMF TEs were highly comparable for isolates from both tissue states. In application, this method effectively detected dietary differences in EMF Fe of a murine feeding study and identified the disease status in a Wilson disease rat model based on drastically increased EMF Cu. In summary, the present method is suitable for future applications, facilitating sample storage and high-throughput analyses of mitochondrial TEs.

**Graphical abstract:**

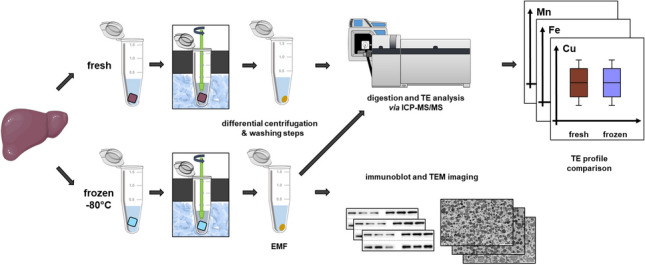

**Supplementary Information:**

The online version contains supplementary material available at 10.1007/s00216-024-05400-y.

## Introduction

Mitochondria are the most crucial organelles for bioenergetic processes and the TEs manganese (Mn), iron (Fe) and copper (Cu) play a central role as cofactors for oxidative phosphorylation within mitochondria [1]. Intra-organelle TE content, however, is often disregarded compared to whole cell, tissue, or serum TE concentrations. Age-associated alterations in liver tissue and serum TE homeostasis are well known [2, 3] as observed in the Interactions of essential trace elements in healthy and diseased elderly (TraceAge) project, which investigates age-specific TE profiles. However, only few analyses have been conducted to investigate the role of mitochondrial TE levels regarding aging, especially from frozen tissue, as there are multiple challenges like cryodamage from tissue storage, required isolation time per sample, and TE quantification in small sample volumes of an EMF. Mitochondria are typically isolated from fresh tissue samples to avoid alterations of the mitochondrial morphology towards the orthodox state upon freezing and thawing [4]. This state features a large matrix but small cristae volume and close proximity of inner and outer mitochondrial membrane (IM, OM) [5]. Further, cryodamage can occur, causing variable membrane permeabilization which impairs oxidative phosphorylation efficiency [6] through the loss of cytochrome c from the intermembrane space [7]. Likewise, if also the inner mitochondrial membrane is ruptured, mitochondrial TE could be lost as the majority of Fe, and Cu is stored in the mitochondrial matrix [8, 9]. Another challenge is the sample throughput, as animal experiments modulating three to five TE can easily surpass 100 animals per experiment [10]. With conventional devices for tissue homogenization such as semi-automated Teflon/glass potter or pump-controlled cell rupture (PCC) systems, approximately 12 min for homogenization and 5 min for cleaning [4] are required, rendering subsequent isolations from fresh tissue for larger numbers of samples difficult.

In order to address the mentioned challenges, we aimed for a quick mitochondria isolation process from as little as 30 to 40 mg frozen liver aliquots with a sufficient yield for TE quantification in the EMF. For that purpose, a Braun Biotech Potter S was customized with a 3D printed quick-change hold for 1.5-mL Eppendorf Tubes® and a quick-grip clamp for autoclavable polypropylene EPPI-pestles (schuett-biotec GmbH), resulting in a simple and convenient homogenization setup implementable to almost any laboratory. The isolation method was confirmed by immunoblotting mitochondrial marker proteins, transmission electron microscopy (TEM) images, and the relative protein yield of the EMF isolated from murine liver aliquots. Further, EMFs from fresh and frozen murine liver tissue and from frozen liver of a rat model for Wilson disease (diseased *Atp7b*^*−/−*^ knockout and healthy *Atp7b*^*+/−*^ control rats), a genetic disease causing hepatic, and in consequence mitochondrial Cu accumulation [11] were compared regarding Mn, Fe, and Cu content, as a proof of concept for the assessment of quantitative TE differences. TEs were quantified via ICP-MS/MS after microwave-assisted low acid digestion with prior validation based on spiked isolation buffer and EU-certified reference material.

## Materials and methods

### Potter customization

**Fig. 1:**
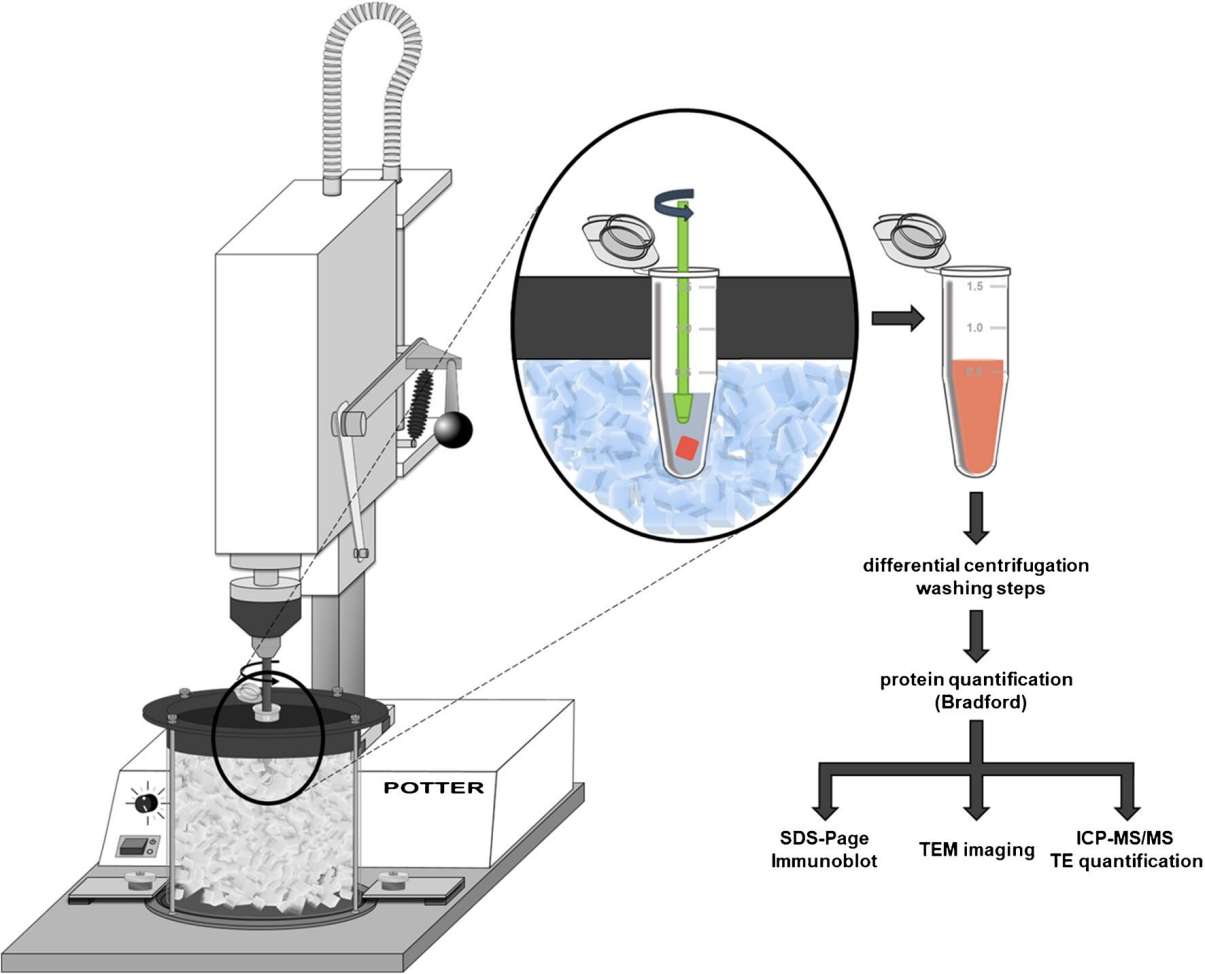
**Schematic customized potter, mitochondria isolation, and analysis workflow**. Potter customized with a 3D printed quick-change hold for Eppendorf Tube® during ice cooled tissue homogenization concentrations. Consecutive analysis workflow of differential centrifugation for mitochondria isolation as well as further downstream analysis is shown

We aimed for a consistent method to isolate an EMF from as little as 30 to 40 mg of frozen liver tissue aliquots in 1.5-mL reaction tubes to increase the throughput and simplify sample storage and later sample handling. A Braun Biotech Potter S (Sartorius Stedim Biotech GmbH) was customized with a 3D printed lid of polylactic acid and a quick-change hold for 1.5-mL Eppendorf Tubes^®^. Additionally, a drill chuck was installed to the potter, to which the quick-grip clamp and the corresponding polypropylene pistil (schuett-biotec GmbH) were attached (Fig. 1).

Chemicals, buffers, and solutions were P.A. grade and are listed in the online resource 1, supplementary Table 1.

### Statement of animal rights, animal housing, and liver preparation

C57BL/6J mice aged 22 or 76 weeks (B6; original strain Jackson Lab) were obtained from in-house breeding and housed under 12-h light/dark cycle with water and food ad libitum. All mice received a rodent standard diet (SD, V1534) or a high-fat-high-carb diet (HFD, EF Bio-Serv F1850) over a period of 8 weeks, both ssniff Spezialdiäten GmbH, Soest, Germany. Dietary TE content is shown in online resource 1, supplementary Table 2. Housing conditions and animal experiments were carried out according to German law on protection of animals, and the experimental protocol was approved by local authorities (Landesamt für Gesundheit und Soziales, Berlin (LAGESO) und Landesamt für Arbeitsschutz, Verbraucherschutz und Gesundheit Brandenburg, Potsdam (LAVG), Germany, approval number: 2347–46-2019). For organ collection, mice were anesthetized. The liver was flushed with PBS via the portal vein, and fresh aliquots were prepared and either directly used for mitochondria isolation or snap frozen in liquid N_2_ and stored at − 80 °C.

Rat experiments were approved by the government authorities of the Regierung von Oberbayern, Munich, Germany, and all animals were maintained under the Guidelines for the Care and Use of Laboratory Animals of the Helmholtz Center Munich. The LPP rat strain, an in-house crossbreed between Long Evans cinnamon rats and Piebald Virol Glaxo rats, was provided by Jimo Borjigin (University of Michigan, Ann Arbor, Michigan, USA). Rats were fed ad libitum on 1314, Altromin Spezialfutter GmbH, Germany (Cu content 13.9 mg/kg), and tap water. Throughout this manuscript, *homozygote Atp7b*^*−/−*^* knockout* (*Atp7b*^*−/−*^ KO) rats represent the defect Cu transporter analogue to a Wilson disease phenotype, and *heterozygote Atp7b*^*+/−*^ rats are used as controls. Until mitochondria isolation, liver tissue aliquots were stored at − 80 °C.

### Mitochondria isolation and protein quantification

The liver aliquots were suspended in 300 µL of ice-cold isolation buffer with bovine serum albumin (BSA) (IP +) and homogenized with 9 strokes of 1 s at 900 RPM in Eppendorf Tubes^®^. Another 300 µL IP + were added to saturate proteases prior to two centrifugation steps at 800 × g (10 min, 4°C) removing the tissue debris. The supernatant was collected and centrifuged at 9000 × g (10 min, 4°C), separating the EMF. After removing the supernatant, the EMF was washed twice with 600 µL BSA free isolation buffer (IP-) and centrifuged again at 9000 × g (10 min, 4°C), before resuspension in 250 µL of IP-. In the 800 × g pellet, the 9000 × g supernatant and the EMF protein content is quantified via Bradford assay, with dilution factors of 200, 80, and 20, respectively. Prior to TE quantification, the protein content of the EMF was normalized to 1 µg/µL with ultra-pure deionized water.

### SDS page and immunoblot

Gels were prepared one day in advance, as indicated (online resource 1, supplementary Table 3). EMF triplicates isolated from liver aliquots originating from a 24-week-old female mouse, fed with the V1534 standard diet, and were diluted 1:4 with 4 × Laemmli with β-mercaptoethanol, and the protein content was adjusted to 1 µg/µL. After heating the samples to 95 °C for 5 min they were stored at − 20 °C. The heating was repeated before gel loading of 5 µg (= µL) protein per sample, along with two replicates of 3 µL PageRuler™ Plus Prestained Protein Ladder (Fisher Scientific) per gel. SDS polyacrylamide gel electrophoresis is done by initially applying 80 V for 40 min and 125 V for 1 h and 45 min followed by wet tank blotting on PVDF membranes for 1 h at 100 V. After Ponceau staining for 1 min and de-staining for 2 times 2 min, membranes were blocked in either 5% non-fat milk powder or 5% BSA in TBS-T for 1 h at room temperature. Overnight, the membranes were incubated with primary antibodies from online resource 1, supplementary Table 4, at 4 °C. HRP-conjugated goat anti-rabbit or goat anti-mouse secondary antibodies (Bio-Rad Laboratories GmbH) were incubated for 1 h at room temperature in 5% non-fat milk powder in TBS-T. Proteins were detected using the Clarity Max™ Western ECL Substrate (Bio-Rad Laboratories GmbH). Freshly prepared 1:2 dilution of luminol/enhancer and peroxide solution were applied to each membrane for 1 min prior to chemiluminescence detection in the ChemiDoc™ MP imaging System (Bio-Rad Laboratories GmbH).

### Transmission electron microscopy pictures

Equivalents of 100 µg protein of the EMF were loaded to 1.5 mL reaction tubes and centrifuged at 9000 × g (10 min, 4 °C). The supernatant was removed, and the reaction tubes were completely filled with 2.5% glutaraldehyde in 0.1 M sodium cacodylate buffer, pH 7.4, and centrifuged again at 9000 × g (1.5 min, 4 °C). Samples were stored at 4 °C and post-fixed in 1% osmium tetroxide, dehydrated with acetone, and embedded in epoxy resin. Ultrathin Sects. (50–60 nm) were cut using Ultracut E (Reichert und Jung) and stained with UranyLess (#DM22409-20; Science Services) and 3% lead citrate (Leica). Images were acquired using a Jeol 1200 EXII electron microscope (Akishima, Tokyo, Japan) at 60 kV, equipped with a KeenViewII digital camera (Olympus, Hamburg, Germany) and processed with the iTEM software package (anlySISFive; Olympus).

### Spike preparation and TE quantification

From freshly prepared IP and element standards (Carl Roth, 1 g/L), two spike stocks were prepared containing either 0.5, 10, and 2.5 mg/L or 0.05, 1, and 0.25 mg/L Mn, Fe, and Cu, respectively. From these stocks, nine spike concentration levels were prepared. Two hundred microliters spike, 100 µL H_2_O_2_, 100 µL HNO_3_, 580 µL H_2_O, and 20 µL of an internal standard containing 500 µg/L Ge and 50 µg/L Rh were subjected to microwave-assisted acid digestion in a MARS 6 microwave digestion system (CEM, Kamp-Lintfort, Germany). The spikes were digested in triplicates per concentration on 5 consecutive days, along with three replicates of 10–22 mg reference material (ERM-BB422 fish-muscle) and four blanks. TEs were quantified via ICP-MS/MS (Agilent ICP-QQQ-MS 8800, Agilent Technologies, Waldbronn, Germany) with working parameters as described in online resource 1, supplementary Table 5. The limit of detection and quantification (LOD and LOQ, respectively) were calculated based on at least ten technical blanks over the five measurement days (at least two per day) with 3σ (standard deviation) for LOD or 10σ for LOQ. The method LOD and LOQ (Table 1) were derived from the LOD and LOQ, considering the sample dilution of 1:5. TE of the EMF with normalized protein content were quantified with the same method as the spikes, by digesting and analyzing equivalents of 200 µg protein (= 200 µL).

**Table 1:** Validation parameters for EMF TE quantification

	^55^Mn	^56^Fe	^63^Cu
Calibration	External	External	External
Calibration mean *r*^2^	0.9999	0.9998	0.9998
Method LOD [µg/L]	0.30	11.8	1.74
Method LOQ [µg/L]	0.55	16.7	2.45
ERM-BB422 FISH MUSCLE reference material			
Reference concentration [mg/kg]	0.368	9.400	1.670
Mean exp. concentration [mg/kg]	0.343	8.488	1.506
Mean recovery [%]	93.2	90.3	90.2
Intraday precision [%]; (*n* = 3)	2.7	4.6	2.6
Interday precision [%]; (*n* = 5)	3.3	5.7	3.1

### Statistical analysis

Statistical analysis was performed in GraphPad™ Prism, version 8.4.3. Data was screened for outliers with the ROUT method. Normal distribution was tested with the D’Agostino and Pearson test. In case of multiple groups, data was analyzed by an ordinary one-way ANOVA with Tukey’s multiple comparisons test, whereas in case of only two groups, a two-sided unpaired *t*-test was performed. Significance was assumed at a *p*-value < 0.05 with *p ≤ 0.05; **p ≤ 0.01, and ***p ≤ 0.001 and trends at 0.05 < *p*-value < 0.1.

## Results

### Validation: TE quantification of spiked and digested isolation buffer

**Fig. 2:**
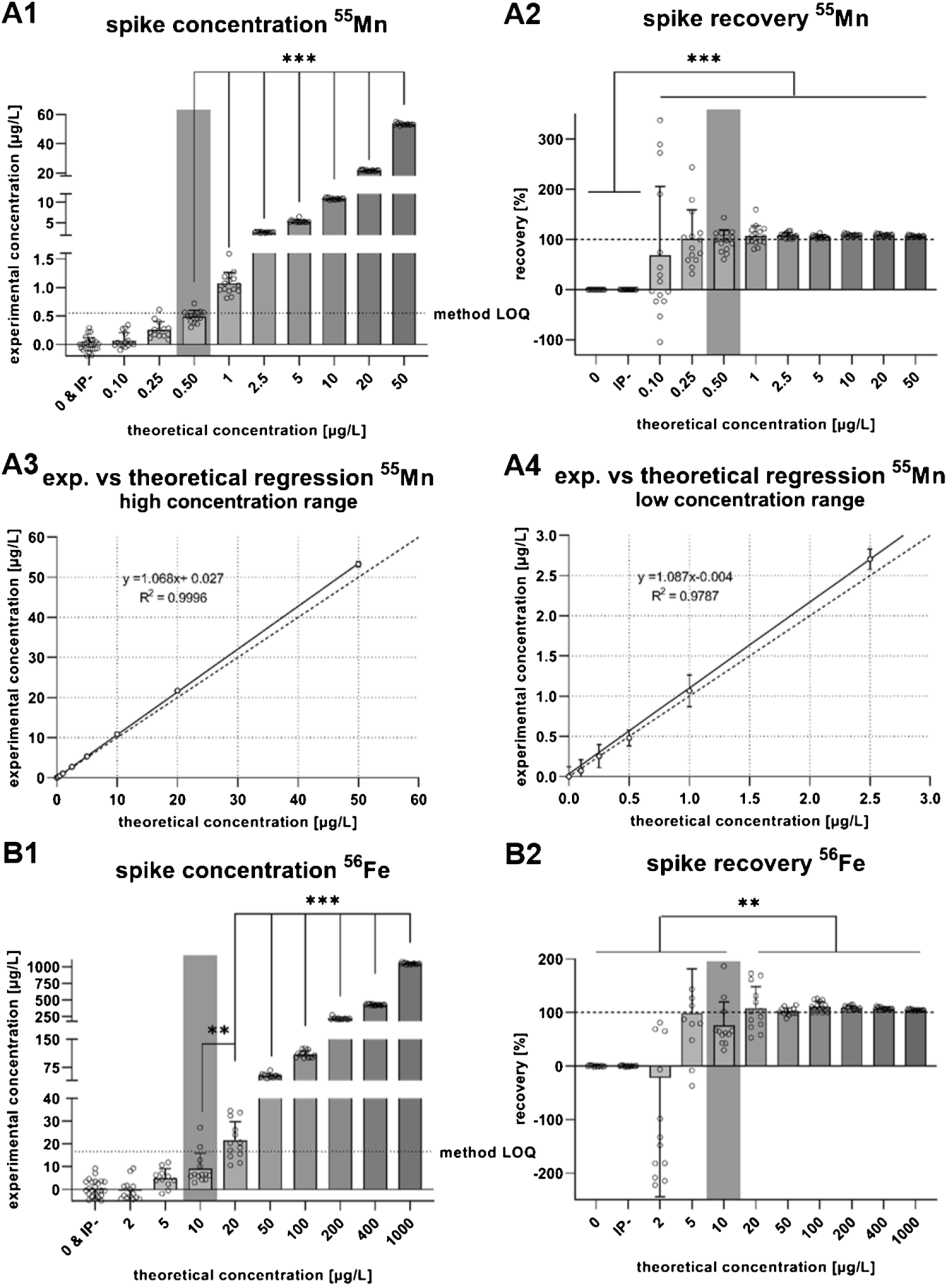

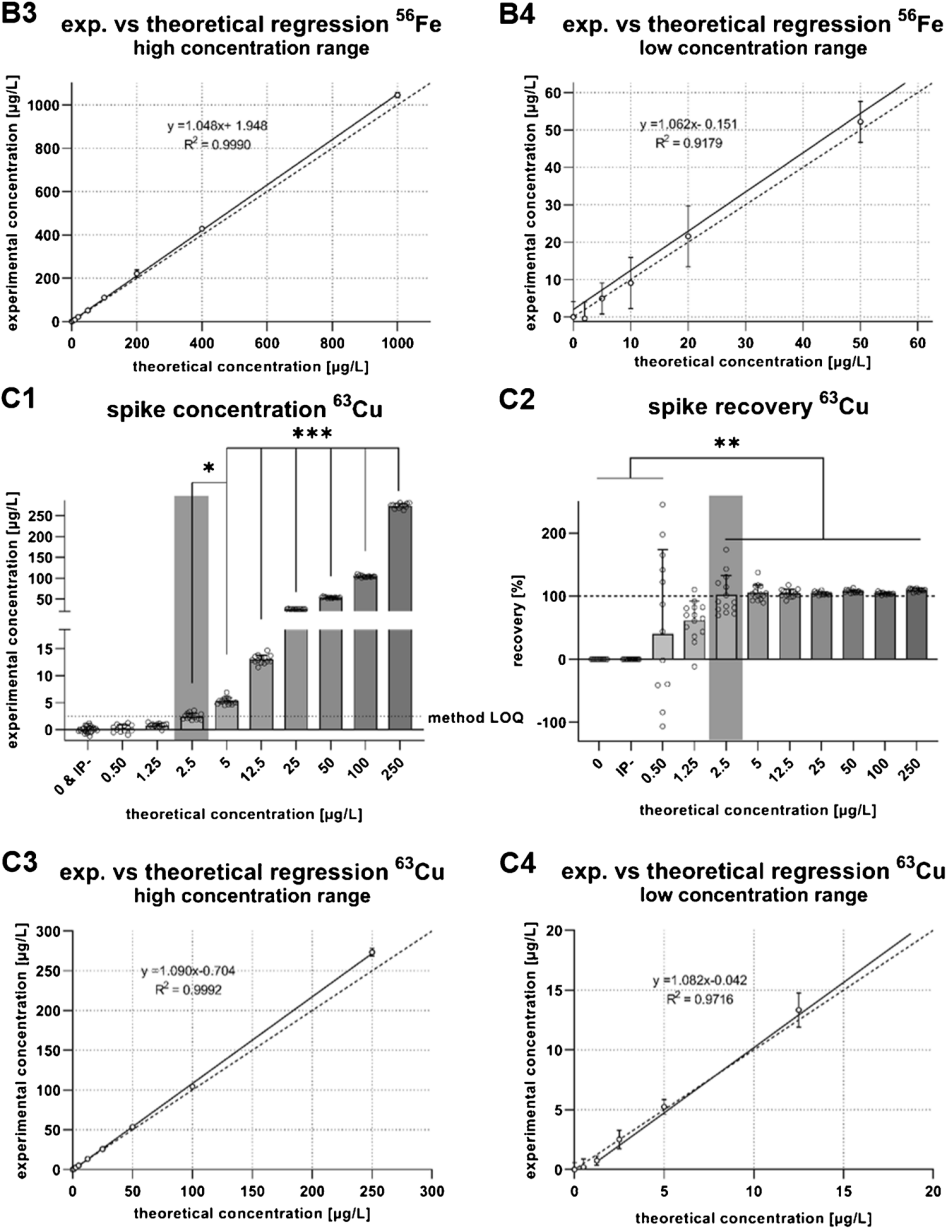
**Mn, Fe, and Cu concentrations, recovery, and simple linear regression of spiked and digested isolation buffer (IP-)**. ^55^Mn (A), ^56^Fe (B) and ^63^Cu (C) spike concentrations (1), recovery of the individual spikes (2), simple linear regression of all spike concentrations (experimental against theoretical) (3) as well as a simple linear regression of spikes of lower concentrations (4). The first spike concentration, significantly distinguishable from water and isolation buffer blanks, is highlighted in gray. Data is shown as mean ± SD with *p ≤ 0.05, **p ≤ 0.01, and ***p ≤ 0.001

For the validation of the TE quantification protocol via ICP-MS/MS, linearity of the calibration curve, method LODs, method LOQs, reference material recovery, and intra- and interday precision based on the reference material were assessed (Table 1). Further, the first spike concentration that was significantly distinguishable from water and isolation buffer blanks was identified as well as the spike recovery. For ^55^Mn, ^56^Fe and ^63^Cu method LODs of 0.3 µg/L, 11.8 µg/L, and 1.74 µg/L and method LOQs of 0.55 µg/L, 16.7 µg/L, and 2.45 µg/L were calculated, respectively (Table 1). The lowest spike distinguishable was 0.5 µg/L, 10 µg/L, and 2.5 µg/L for ^55^Mn, ^56^Fe, and ^63^Cu (Fig. 2: A1, B1, and C1). These spike concentrations also mark the threshold for decreasing variance in the spike recovery (Fig. 2: A2, B2, and C2). Graphs labeled with 3 (all spikes) and 4 (lowest five spike concentrations) reflect the experimental spike concentrations compared to the theoretical ones. Correlation coefficients (*R*^2^) were 0.9996, 0.999, and 0.9992 (Fig. 2: A3, B3, and C3) for all concentrations of ^55^Mn, ^56^Fe, and ^63^Cu, respectively, and the slopes were 1.068, 1.048, and 1.09. Similar slopes along with lower *R*^2^ of 0.978, 0.918, and 0.972 (Fig. 2: A4, B4, and C4) for ^55^Mn, ^56^Fe, and ^63^Cu were observed for the low concentration range.

### Mitochondrial marker proteins, contamination marker proteins, and TEM pictures from EMF of frozen murine liver tissue

**Fig. 3:**
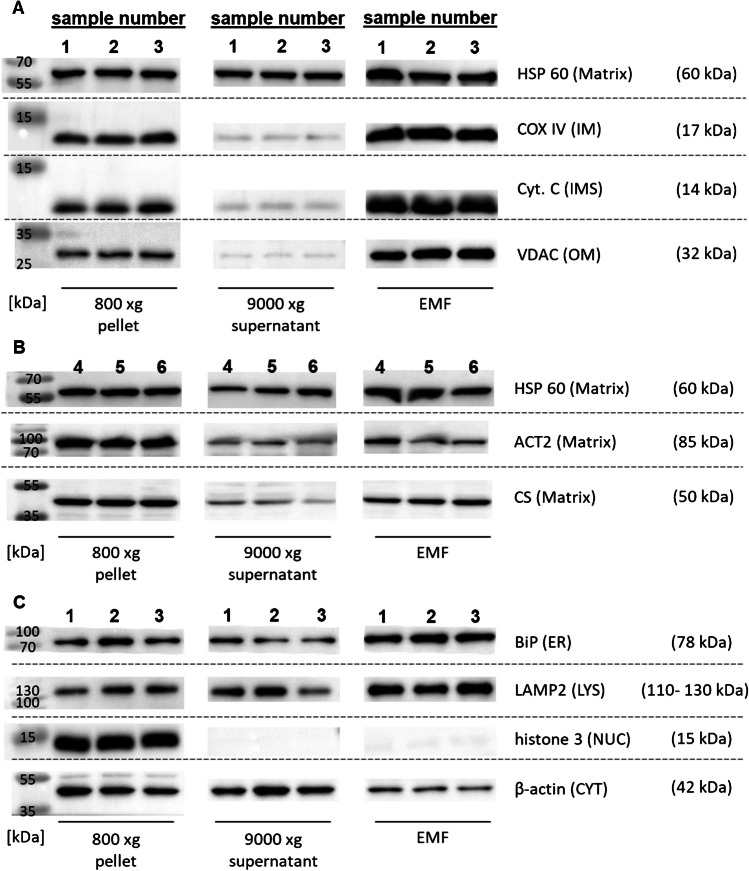
**Immunoblots of mitochondrial proteins and contamination markers from frozen murine liver samples**. Immunoblots of 5 µg protein separated by SDS gel electrophoresis of the 800 × g pellet, 9000 × g supernatant, or the EMF. Samples were blotted in replicates per lane (1, 2, 3 during one blot and 4, 5, and 6 as a follow-up for matrix proteins). Mitochondrial marker proteins: HSP60 (heatshock protein 60, mitochondria matrix); COX IV (cytochrome c oxidase subunit 4, inner mitochondria membrane); Cyt.C (cytochrome C, intermembrane space); VDAC (voltage-dependent anion channel, outer mitochondria membrane) (**A**). Mitochondrial matrix marker proteins: HSP60; ACT2 (aconitase 2); CS (citrate synthase) (**B**). Organelle contamination marker proteins: BiP (binding immunoglobulin protein, endoplasmic reticulum); LAMP2 (Lysosome-associated membrane protein 2, lysosomes); histone 3 (nucleus); β-actin (cytoskeleton) (**C**)

Immunoblotting of mitochondrial marker proteins and marker proteins of other cell organelles of the 800 × g pellet, the 9000 × g supernatant and the EMF (Fig. 3A to C) shows semi-quantitative increase of the voltage-dependent anion channel (VDAC), cytochrome C (Cyt. C), and cytochrome c oxidase subunit 4 (COX IV) from the tissue pellet to the EMF. Hardly any mitochondrial proteins are apparent in the 9000 × g supernatant. In contrast, the mitochondrial matrix protein HSP60 is evenly distributed across the three fractions (Fig. 3A). This distribution is also observed in another set of triplicates for HSP60 and to a lower degree for aconitase 2 (ACT2) (Fig. 3B). In contrast to the other matrix proteins, citrate synthase (CS) showed lower concentrations in the 9000 × g supernatant, compared to the respective EMF (Fig. 3B). Immunoblotting of marker proteins of other cell organelles shows an increase in proteins associated to the endoplasmic reticulum (ER) and lysosomes from the 800 × g pellet to the EMF. In contrast to the proteins of the mitochondrial membranes, ER and lysosomal proteins are also present in the 9000 × g supernatant (Fig. 3C). Nuclear proteins are only detected in the 800 × g pellet, but not in the 9000 × g pellet or the EMF. A reduced content of the cytoskeletal protein β-actin is observed for the EMF compared to the even distribution in the 800 × g pellet and 9000 × g supernatant.

**Fig. 4:**
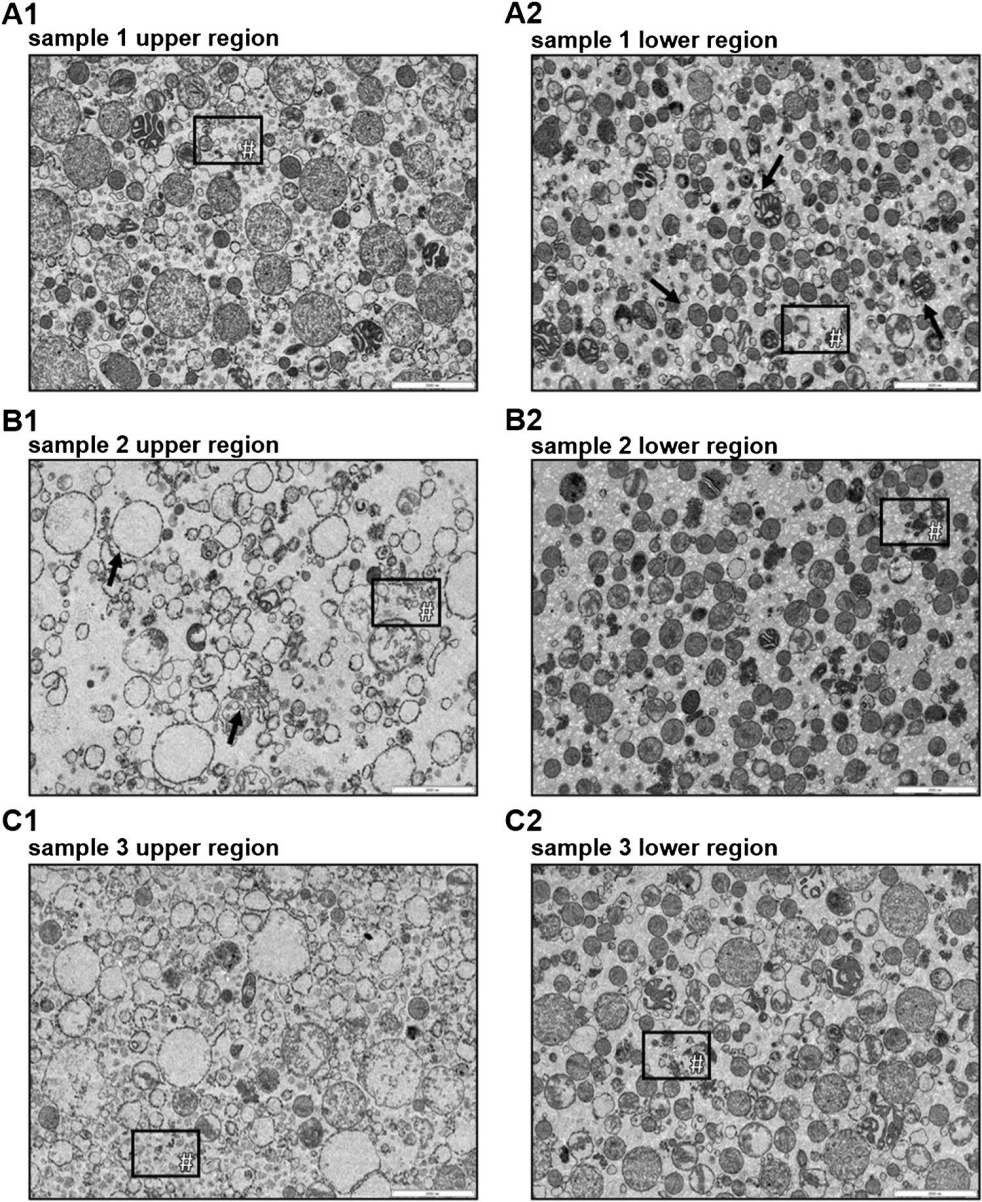
**Transmission electron microscopy (TEM) pictures of selected EMFs with differing integrity**. Representative TEM pictures of EMFs isolated from liver tissue aliquots of three different animals. Samples represent 100 µg EMF protein with a scale bar of 2000 nm (bottom right corner). Same numbers indicate pictures from the same EMF. “Upper” or “lower region” refer to the vertical cut (top view). Exemplary features of the preserved areas (intact inner and outer mitochondrial membrane and preserved matrix) are indicated by arrows (sample 1 lower region). Features of impaired areas (damaged membranes and empty organelles) are indicated by arrows (sample 2 upper region). Debris is indicated # labeled boxes

TEM pictures of 100 µg EMF protein showed isolated mitochondria (Fig. 4). Areas of impaired (column 1, upper region) and preserved (column 2, lower region) integrity within individual samples were revealed (Fig. 4), depending on the vertical position of cutting the resin embedded pellet. Cuts in upper regions of the EMF pellet showed predominantly impaired integrity featuring empty organelles and damaged membranes (Fig. 4: A1, B1, and C1), while preserved organelles, located in the lower region of the pellet, show mainly intact inner and outer mitochondrial membrane (IM and OM) as well as conserved mitochondrial matrix inside (Fig. 4: A2, B2, and C2). Differences are observed for both, the impaired and the preserved regions, as for the impaired areas no mitochondrial matrix is contained in Figure B1 and C1, compared to Figure A1. Preserved areas contain more condensed and compact mitochondria in Figure A2 and B2, compared to C2 with intact membranes and conserved matrices. Both, impaired and conserved areas contain smaller debris and empty vesicular structures indicated by # labeled boxes.

### Method application: comparing protein yield of EMFs from frozen vs. fresh murine liver tissue

**Fig. 5:**
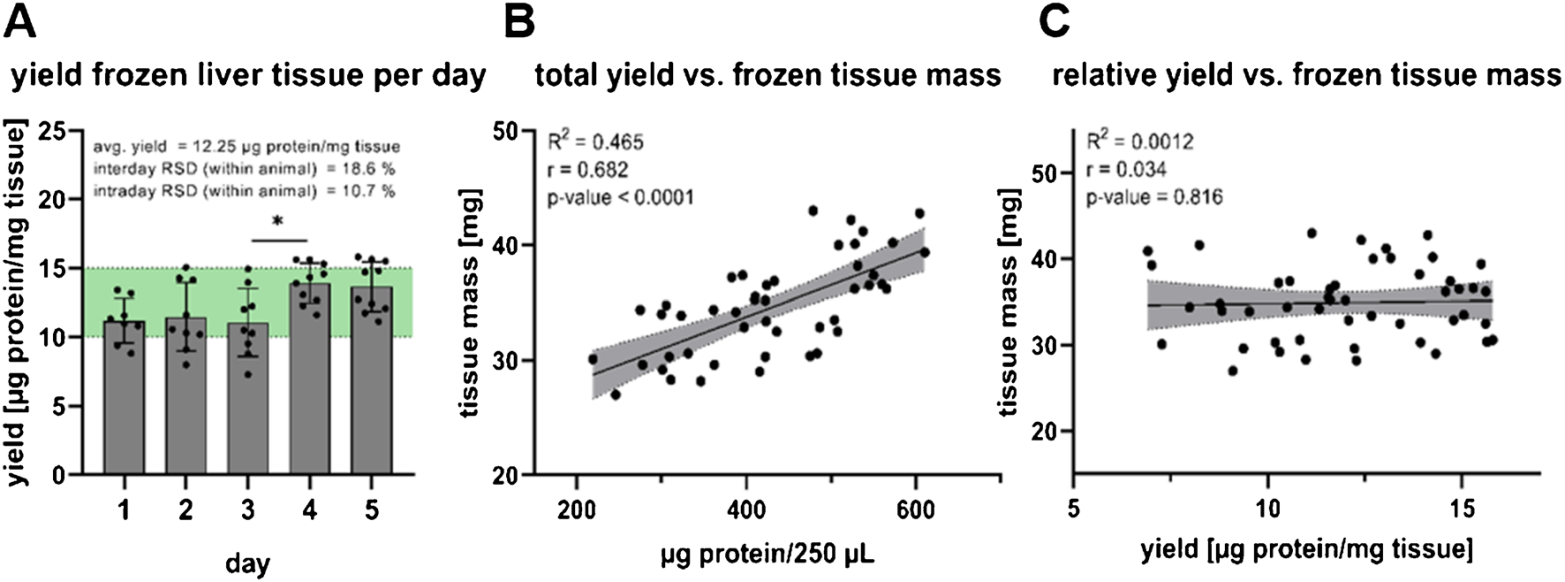
**Total protein yield from frozen murine EMFs and correlation of total and relative yield with the homogenized tissue mass**. Relative EMF protein yield in μg/mg frozen murine liver tissue homogenized, with the targeted yield range in green (**A**). Correlation between the absolute (**B**) or relative (**C**) EMF protein yield with the homogenized tissue mass. Pearson correlation and ordinary one-way ANOVA with Tukey’s multiple comparisons test with *p ≤ 0.05, **p ≤ 0.01, and ***p ≤ 0.001

The described method was initially applied to a subset of 50 frozen liver tissue aliquots from five different animals aged 76 weeks. Over the course of 5 days, EMFs were isolated from two replicates per animal. The EMF protein content was quantified and normalized, and the trace elements were quantified as described. The average relative protein yield per day was 12.25 µg/mg tissue within the predefined range of 10 to 15 µg/mg tissue. This range was set in advance of the experiment, to ensure sufficient sample material for the performance of immunoblots, sample preparation for TEM pictures, and TE analysis from one isolated EMF. A significant difference in yield was found comparing days 3 and 4 (Fig. 5A). Intraday- and interday precision of the relative protein yield were 10.7% and 18.6% within each animal, respectively (Fig. 5A). Additionally, the supernatant protein content obtained from the EMF isolation of the frozen liver tissue samples was quantified (online resource 2, supplementary Fig. 2) and compared in groups based on a 10 µg/mg tissue threshold for the corresponding EMF, representing the lower limit of the targeted yield range. EMF isolated with a protein yield ≥ 10 µg/mg tissue also had significantly more protein in the corresponding supernatant after centrifugation at 9000 × g (online resource 2, supplementary Fig. 2).

For the frozen tissue, a strong correlation between the homogenized tissue mass and the total protein yield of the EMF was observed (Fig. 5B), but not for the relative yield with the homogenized tissue mass (Fig. 5C). For comparison, the same method was subsequently applied to a small subset of 10 fresh liver tissue aliquots from five different animals. EMFs isolated from fresh murine liver tissue samples had a lower yield with an average relative EMF protein content of 8.9 µg/mg tissue (online resource 2, supplementary Fig. 1A), slightly below the predefined optimal range. No significant correlation could be observed for either the total or the relative EMF protein yield with the corresponding homogenized tissue mass (online resource 2, supplementary Fig. 1B and C).

### Method application: comparing EMF and total liver TE concentration from frozen vs. fresh murine tissue

**Fig. 6:**
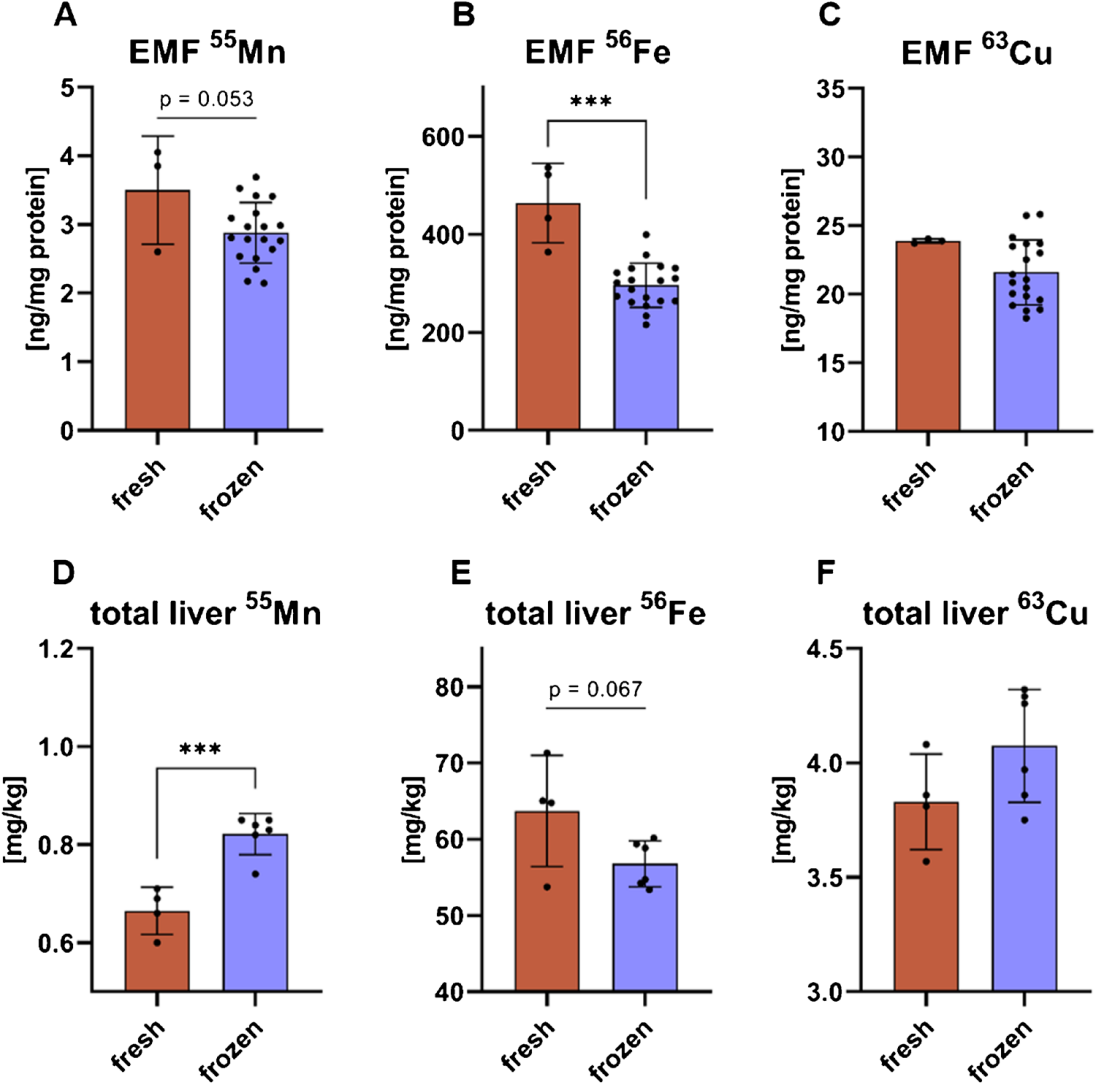
**Comparison of Mn, Fe, and Cu content of EMFs and the corresponding total tissue of frozen against fresh murine liver tissue**. EMF Mn (**A**), Fe (**B**), and Cu (**C**) concentrations, isolated from fresh (red) and frozen (blue) murine liver tissue. Total hepatic Mn (**D**), Fe (**E**), and Cu (**F**) concentrations per kg wet weight of fresh (red) and frozen (blue) murine liver tissue. Statistical analysis via unpaired *t*-test with *p ≤ 0.05, **p ≤ 0.01, and ***p ≤ 0.001

In order to investigate a potential loss of EMF TEs and also carry-over from the whole liver tissue during isolation, EMFs and total liver TEs from fresh and frozen liver tissue aliquots were quantified. Thereby, fresh liver tissue aliquots of two animals were compared in duplicates against frozen ones. Both animal groups received the Serv F1850 (HFD) with the lower TE concentration (online resource 1, supplementary Table 2). EMF Mn and Cu did not differ between fresh and frozen isolates (Fig. 6A and C), while Fe was lower in the EMF isolated from frozen tissue samples (Fig. 6B). The total hepatic TE concentrations of Cu was similar between fresh and frozen aliquots (Fig. 6F), but total hepatic Mn concentrations were increased in the frozen tissue aliquots compared to the fresh aliquots (Fig. 6D).

### Method application: dietary effects on EMF and total TE concentration of frozen murine liver samples

**Fig. 7:**
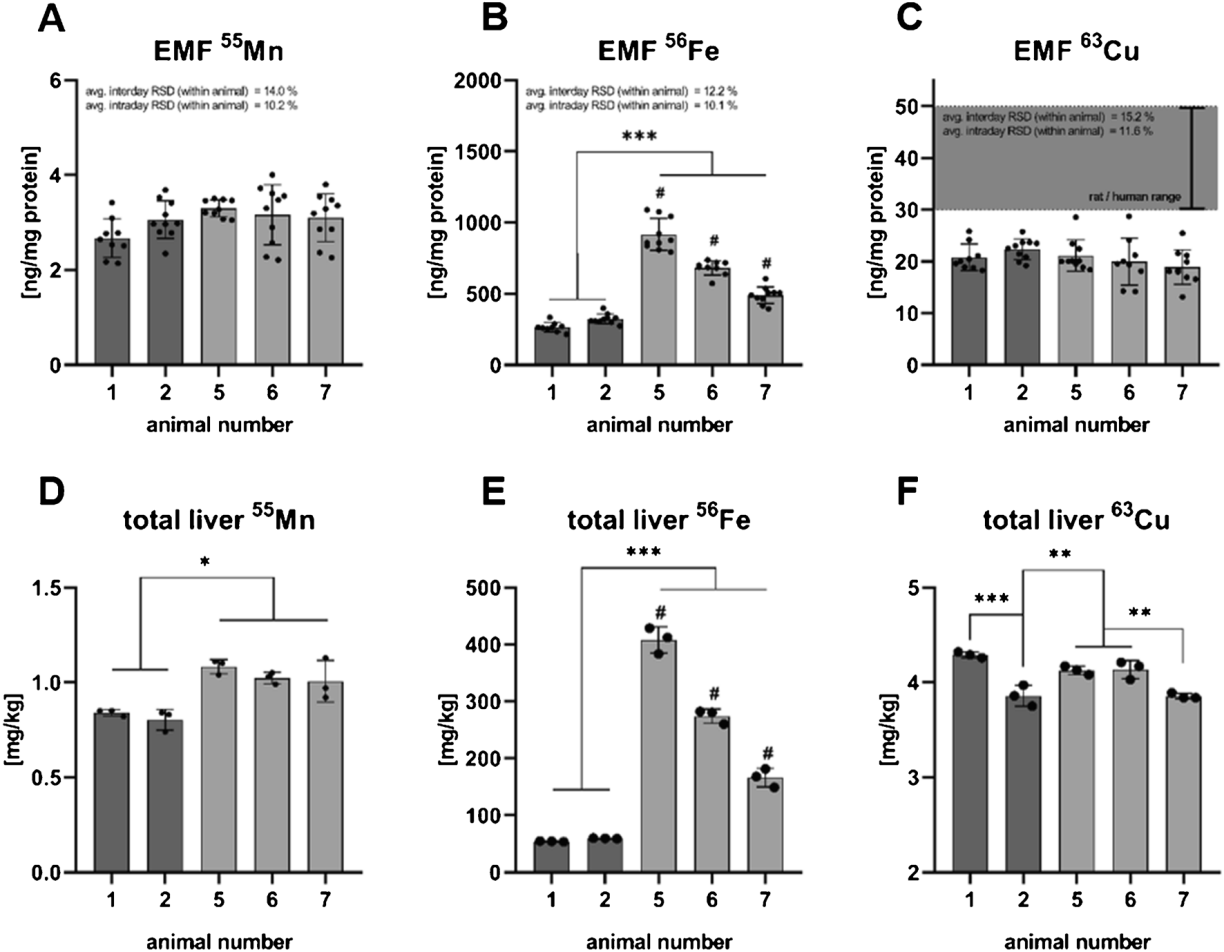
**Mn, Fe, and Cu content of EMFs and the corresponding total tissue from frozen murine liver tissue**. Relative Mn (**A**), Fe (**B**), and Cu (**C**) content in ng/mg of EMF protein. Total hepatic Mn (**D**), Fe (**E**), and Cu (**F**) concentration in mg/kg tissue. Mean concentrations and the RSD in % per group indicated in white. # indicates ***p ≤ 0.001 significance between labeled animals. High-fat diet indicated in dark gray, and standard diet indicated in light gray. Statistical analysis via ordinary one-way ANOVA with Tukey’s multiple comparisons test with *p ≤ 0.05, **p ≤ 0.01, and ***p ≤ 0.001

Mn and Cu concentrations of the EMF were constant for all animals with an average of 3.06 and 20.6 ng/mg, respectively (Fig. 7D and F). Significant differences were found for EMF Fe with 1.5 to 3 times increased Fe concentrations for animal 5, 6, and 7 (V1543) in the EMF compared to animals 1 and 2 (Bio-Serv F1850 diet), with an average of 530.3 ng/mg EMF protein (Fig. 7E). Similar, total liver Mn and Fe were also significantly increased for animals 5, 6, and 7 compared to animals 1 and 2 (Fig. 7A). No differences were observed for total hepatic Cu (Fig. 7C). The fold-change magnitude for the total hepatic Fe between animals with different diets was on average 2 times the fold-change of EMF Fe (Table 2). Comparison of the fold-change of the total TEs and the EMF content showed no differences for Mn and Cu between animals with either the EF Bio-Serv F1850 or the V1534 diet.

**Table 2:** Fold-change comparison of total liver and EMF TEs between animals 1 and 2 (Serv F1850 HFD) against 5, 6, and 7 (V1534 SD)

	Serv F1850 (HFD, animals 1 and 2) vs. 1534 V (SD, animals 5,6, and 7)					
	Bulk 1.2 vs. 5	EMF 1.2 vs. 5	Bulk 1.2 vs. 6	EMF 1.2 vs. 6	Bulk 1.2 vs. 7	EMF 1.2 vs. 7
^55^Mn	1.3	1.2	1.2	1.1	1.2	1.1
^56^Fe	7.2	3.1	4.8	2.2	2.9	1.7
^63^Cu	1.0	1.0	1.0	0.9	0.9	0.9

### Method application: comparing EMF and total liver TE concentration from a Wilson disease rat model

To confirm the disease status of our model, we conducted TE analysis within EMF and total liver tissue. No significant differences in mitochondrial or total liver Mn and Fe content were observed between healthy and Wilson disease rat models (online resource 2, supplementary Fig. 3A, B, D, and E). An increase was observed in EMF Cu by a factor of 18.2 (online resource 2, supplementary Fig. 3C) and total liver Cu by a factor of 29.2 (online resource 2, supplementary Fig. 3F) in the diseased animals compared to their healthy counterparts, confirming the disease status.

## Discussion

Prior to the establishment and adaptation of the isolation method, optimal HNO_3_ concentrations for digestion (data not shown) as well as detection and quantification limits were identified with validation experiments analyzing matrix-matched spikes. The first significantly distinguishable concentration from unspiked isolation buffer was identified with 0.5 µg/L, 10 µg/L, and 2.5 µg/L for Mn, Fe, and Cu, respectively (Fig. 1: A1, B1, and C1). With 0.55 µg/L, 16.7 µg/L, and 2.45 µg/L (Table 1), the calculated method LOQs further confirmed the limits of the present digestion and quantification method. Linearity of the calibration curves was sufficient, as well as the correlation coefficients of the spikes comparing the experimental values against the theoretical ones with > 0.999 for all three elements. The *R*^2^ of the lowest five concentrations (Fig. 1: A4, B4, and C4) was slightly reduced but still sufficient. Therefore, linearity can be assumed across the concentration range. The slopes, representing spike recovery, were as follows: Mn 1.068, Fe 1.048, and Cu 1.090, which are comparable to past validations for other biological samples [12] and were almost unchanged compared to the lowest spikes. Recovery rates of the certified reference material ranged from 90.2 to 93.3% with intraday variance < 5% and interday variance < 6% for Mn, Fe, and Cu (Table 1). This indicates, that in comparison to other microwave-assisted acid digestion protocols, a concentration of 10% (v/v) HNO_3_ is sufficient to digest the EMF samples, whereas other approaches use up to 50% (v/v) HNO_3_ for the initial digestion [13]. Such low acid concentrations eliminate the need for further dilution and allow direct subjection to the ICP-MS/MS. In consequence, lower concentrations can be detected and less sample is needed.

After identifying the analytical limits, the potter was customized to directly operate in Eppendorf Tubes®, lowering the time for tissue homogenization down to less than 30 s per sample which, as initially stated, drastically improves the throughput compared to other methods [4]. This adaptation was required. As initially stated, within the TraceAge project, animal experiments easily involve up to 100 animals [10, 14]. This is primarily due to the fact that dietary or age-related changes in TE homeostasis in a physiological range exhibit smaller effect sizes compared to genetic defects, like Wilson disease. Consequently, a higher sample size is required to reach statistical significance. As a result, the EMF isolation method was adapted to increase the throughput.

Immunoblotting served as a first confirmation step for our isolation method and was compared with TEM pictures of the EMF, the gold standard for integrity and contamination assessment [4]. The semi-quantitative increase of VDAC, Cyt. C, and COX IV (Fig. 3A) indicates a concentration of the proteins located at the outer and inner mitochondrial membrane or the intermembrane space (IMS), especially with almost no mitochondrial membrane-associated proteins detected in the 9000 × g supernatant (Fig. 3A). This is comparable with results from Schmitt et al*.* [4, 15] confirming the isolation of mitochondria from frozen mouse liver. In contrast, the uniform distribution of the matrix protein HSP60 across all fractions, with only slight increase in the EMF, could indicate storage-induced cryodamage which could cause leakage of proteins and TEs contained in the mitochondria during the isolation process. Consequently, additional matrix proteins like aconitase 2 (ACT2) and citrate synthase (CS) should follow the same pattern as HSP60. As this was not observed (Fig. 3B), a potential explanation could be different antibody sensitivities and incubation requirements (online resource 1, supplementary Table 4). Also, as there is less CS in the 9000 × g supernatant compared to the EMF, it seems likely that there is only marginal damage and consequent leakage from the mitochondrial matrix. The concentration of the contamination marker binding immunoglobulin protein (BiP), as indicator for the ER, as well as the lysosome-associated membrane protein 2 (LAMP2) in the EMF are expected, since density gradient centrifugation is required for the separation of ER or lysosomal contaminations due to their close association or similar physical properties, respectively [16, 17]. However, density gradient centrifugation is only needed to generate a highly pure mitochondrial fraction but significantly slows down the sample preparation process which denies the higher throughput we aimed for. As opposed to ER and lysosomal contamination and to the results from Schmitt et al*.*, no nuclear contaminations indicated by histone 3 (H3) were detected in the 9000 × g supernatant or EMF.

Findings from the present immunoblots are in accordance with the TEM pictures, which further confirm successful mitochondria enrichment from frozen samples (Fig. 4). Areas with impaired (Fig. 4: A1, B1, C1) and preserved mitochondrial integrity (Fig. 4: A2, B2, C2) were observed within the same samples. In line with the stronger concentration of OM, IMS, and IM proteins, compared to the matrix proteins, this reinforces the hypothesis of partial cryodamage due to tissue storage at − 80 °C. Further, in most of the samples, mitochondria featured the orthodox state as exemplarily shown in Fig. 4C, which is described as a known, structure altering effect caused by freezing and thawing [4]. Arguably, this renders most functional follow-up experiments impossible, but since most of the OM and IM are uninterrupted in the areas of preserved integrity, it can be assumed that the TEs are preserved in the organelle, which is crucial for quantification of mitochondrial TEs. In order to further investigate potential leakage or even mitochondrial integrity, also the protein content of the EMF supernatant was quantified (Figure S1) and normalized to the homogenized tissue mass. Initially, a leakage-dependent increase in supernatant protein concentrations for corresponding EMFs with a yield below 10 µg/mg tissue was hypothesized. As the opposite was observed, leakage seems even more unlikely, with the supernatant protein content being more indicative for the overall homogenization efficiency, rather than mitochondrial integrity.

Application of the described method to a subset of liver tissue aliquots revealed consistent protein yield in the EMF of 12.25 µg protein per mg homogenized tissue (Fig. 5A). As only the total, but not the relative EMF protein yield correlates with the tissue mass homogenized (Fig. 5B, C) a constant, reproducible yield of mitochondrial protein per mg tissue can be assumed for the range of 30 to 40 mg tissue per sample. EMF TE quantification showed sufficient within animal intra- and interday precision from 10.2 to 11.6% and 12.2 to 15.2%, respectively.

To investigate if there is a potential loss of TEs from the EMFs from frozen tissue, only the TE content of the EMFs isolated from mice receiving the EF Bio-Serv F1850 (HFD) diet containing lower amounts of TEs (Fig. 6) was compared against EMF TEs from fresh murine liver tissue, receiving the same dietary intervention. Also, the total hepatic TE content of both groups was quantified to exclude influences of the total hepatic TE content on the EMF TE concentration. With 3.29 ng per mg EMF protein for Mn and 23.9 ng/mg EMF protein for Cu (Fig. 6A and C), the EMF TE content is highly similar to those isolated from frozen murine liver. Interestingly, the EMF Fe content in isolates from fresh tissue was higher compared to isolates from frozen tissue (Fig. 6). While no significant statistical difference could be observed (Fig. 6E), there is a trend (*p* = 0.067) for increased total liver Fe content in the fresh tissue samples compared to the frozen ones. Therefore, it is likely that differences in EMF Fe reflect the total tissue concentration, but not freezing and consequently membrane damage-associated loss of Fe, due to the interdependence of mitochondrial Fe homeostasis on both cytosolic and mitochondrial concentrations [18]. In contrast, the lower Mn concentration in the fresh total liver tissue compared to the frozen was not observed for the EMF (Fig. 6A and D), which instead showed a trend of elevated Mn in the EMF from fresh tissue. Considering the limited understanding of Mn uptake mechanisms into mitochondria, aside from the proposed transport via mitoferrin [19], this could underscore a tightly regulated Mn homeostasis within these organelles. Especially, since Mn is discussed as a “mitochondrial life-death switch” [20] due to its dual role, when it accumulates in mitochondria beyond physiological levels, it promotes H_2_O_2_ production, while under normal conditions, it has antioxidative purposes as a cofactor for superoxide dismutase 2 (SOD2).

No differences in EMF TEs with different diets from frozen murine liver tissue were found for Mn and Cu (Fig. 7A and C), which is unexpected for Mn, as the V1534 contains more than three times the Mn of the EF Bio-Serv F1850 diet. In contrast, differences in EMF Fe were detected with 1.7- to 3.1-fold increased Fe content in the present EMFs of animals receiving the V1534 diet (Fig. 4B; Table 2), which is in line with the almost threefold higher Fe content of the V1534 diet. As Cu levels were comparable between the diets, no differences between the EMFs from animals with different diets were expected. Further, a dietary effect on EMF Mn and EMF Cu can be excluded as all fold-changes comparing the two diets regarding the total or EMF TE content, ranged from 0.9 to 1.3 (Table 2) for Mn and Cu. Noticeably, with an average of 20.6 ng per mg EMF protein, the Cu content is only 50% of what was previously reported for rats and humans [11] (Fig. 7C, reference range indicated in gray). However, considering that the range of 30 to 50 ng/mg mitochondrial protein is derived from highly purified rat mitochondria [21–23], it is likely that the difference is caused by the lower purity of the present EMFs.

Bulk analyses showed differences in total hepatic Cu between two groups receiving different diets (Fig. 7F) and could be caused by intra-animal variance as similar total Cu liver concentrations are known [2, 13] and no drastic dietary effects could be concluded. In contrast, dietary differences for Mn and Fe were observed in the liver tissue (Fig. 7D and E). While this complements the changes in EMF Fe and further supports the assumed time dependency of Fe accumulation in the mitochondria based on different diets, it does not apply for EMF Mn. A possible explanation could be the two to three times higher dietary content of Fe compared to Mn in the EF Bio-Serv F1850 diet, considering shared import pathways via the mitochondrial uniporter complex and mitoferrin orthologs for Mn and Fe [24] which might be predominantly used for Fe. A carry-over from total tissue TEs into the EMF during the isolation process for the EMF from frozen murine liver is unlikely, as this would require a similar fold-change in total hepatic and EMF Fe comparing animals receiving the 1534 V SD or Serv F1850 diet. With fold-change pairs of 7.2 and 3.1, 4.8 and 2.2, and 2.9 and 1.7 (Table 2), the fold-change of liver TEs is almost twice the change of the EMF, rendering a carry-over unlikely.

In addition, analysis of the EMF TEs from *Atp7b*^*−/−*^ KO and the healthy *Atp7b*^+/−^ control rats reinforce the principle of the present isolation method. While no alteration in EMF and total hepatic Mn and Fe were observed (online resource 2, supplementary Fig. 3A, B, D, E), the EMF Cu content of the *Atp7b*^*−/−*^ KO rats of 878 ng/mg EMF protein was on average 18.2 times higher than from the Atp7b^+/−^ control rats with 48.2 ng/mg EMF protein. EMF Cu concentrations of the *Atp7b*^+/−^ control rats also confirmed the already discussed range of 30 to 50 ng/mg EMF protein. The 18.2-fold increase of hepatic Cu is comparable to human Wilson disease patients in which concentrations are reported to be elevated by a factor of 5 to 20 [25]. The same applies for the total hepatic Cu concentrations of the diseased *Atp7b*^*−/−*^ KO and the healthy *Atp7b*^+/−^ control rats, with a fold-change of 29.2, confirming the disease status especially considering the similar dietary Cu levels ranging from 12 to 15 mg/kg (online resource 1, supplementary Table 2) all animals received.

## Limitations and outlook

While showing a successful mitochondria isolation with comparable TE concentrations in EMFs isolated from frozen and fresh liver tissue samples, the present method is most likely not suitable for less homogeneous tissues containing more connective tissue, such as the muscle, heart, or lung, as the homogenization conditions are relatively gentle. 

With a total volume of 1 mL of sample after digestion, parallel analyses for elements requiring gas change in the collision reaction cell of the ICP-MS/MS, such as Se, are challenging and require an additional EMF isolate from the same tissue. The quantification of Zn was discarded due to high and not stable background signals from the isolation buffer in preliminary analyses (data not shown). However, for future analyses, the quantification limits of the present method allow for further sample dilution, hence an increased sample volume and analyses of elements requiring an O_2_ mass shift. 

Especially for the comparison of fresh tissue against the frozen samples, larger sample sizes for fresh tissue EMF isolation and TE quantification (Fig. 6) are required. This could equalize the effects currently observed. However, without an in-house animal facility present, the access to fresh tissue samples is limited which is why the promising initial insights require further confirmation. Additional insight into mitochondrial Fe and Mn homeostasis could be gained by gene expression studies and immunoblotting. Potential targets could include the mitochondrial calcium uniporter, which is discussed to be involved into Mn uptake in the mitochondria [19] but also Fe-S cluster protein mitoferrin, considering the transport and storage of Fe from the cytosol [26, 27] into the mitochondria. In a next step, the present method can be applied to various feeding studies within the TraceAge project to further explore the role of mitochondrial TEs in the aging process.

## Conclusion

We developed a robust method for mitochondria isolation from as little as 30 to 40 mg frozen murine liver tissue and validated the isolation process as well as the subsequent sample preparation and TE quantification via ICP-MS/MS. The isolation process allows sample storage prior to the isolation, a fast and high-throughput of samples during the isolation and the storage of the isolates until TE quantification, making the present workflow time efficient and convenient for routine applications. EMF TE profiles from frozen murine liver of animals receiving a higher Fe content via diet showed an increase in EMF Fe, compared to animals receiving a diet containing lower Fe. This effect progressed from the total liver tissue into the EMF during an 8-week dietary intervention. Furthermore, comparison of TE profiles between frozen and fresh tissue samples showed only minimal differences, affirming the present method of mitochondria isolation with consecutive TE analysis for frozen liver tissue samples. Additionally, the TE profiles of EMFs isolated from healthy and diseased *Atp7b*^+/−^ control and *Atp7b*^*−/−*^ KO rats, showed an 18.2-fold increased EMF Cu for the diseased rats, serving as proof-of-concept samples. By enabling both, the identification of drastic mitochondrial TE alterations caused by a genetic disorder such as Wilson disease, but also more delicate effects from dietary interventions, the present method is a valuable tool for future high-throughput mitochondrial isolation and TE quantifications from liver tissue.

### Supplementary Information

Below is the link to the electronic supplementary material.

Supplementary file1 (DOCX 23 KB)

Supplementary file2 (DOCX 378 KB)

## Data Availability

Data of the present study are available upon reasonable request from the corresponding author.
