# Supplementary material for: Subzero project: comparing trace element profiles of enriched mitochondria fractions from frozen and fresh liver tissue
Source: Anal Bioanal Chem. 2024 Jul 3;416(20):4591–604. doi: 10.1007/s00216-024-05400-y (PMC11294433; doi:10.1007/s00216-024-05400-y)
Supplement: Supplementary file 1 — Supplementary file1 (DOCX 23 KB) [file 216_2024_5400_MOESM1_ESM.docx]

**Article Title:**

Sub Zero Project: Comparing trace element profiles of enriched mitochondria fractions from frozen and fresh liver tissue

**Jounral Name:**

Analytical and Bioanalytical Chemistry

**Author Names:**

Tom Heinze^1,2^, Franziska Ebert^1^, Christiane Ott^2,3^, Judith Nagel^4^, Carola Eberhagen^5^, Hans Zischka^4,5^, Tanja Schwerdtle^1,2,6, *^

^1^Institute of Nutritional Science, Department of Food Chemistry, University of Potsdam, Nuthetal, Germany ^2^TraceAge – DFG Research Unit on Interactions of Essential Trace Elements in Healthy and Diseased Elderly (FOR 2558), Berlin-Potsdam-Jena-Wuppertal, Germany
^3^Department of Molecular Toxicology, German Institute of Human Nutrition, Nuthetal, Germany
^4^Institute of Toxicology and Environmental Hygiene, Technical University Munich, School of Medicine and Health, München, Germany
^5^Institute of Molecular Toxicology and Pharmacology, Helmholtz Munich, München, Germany
^6^German Federal Institute for Risk Assessment (BfR), Berlin, Germany

*Corresponding author: [tanja.schwerdtle@uni-potsdam.de](mailto:tanja.schwerdtle@uni-potsdam.de)

orcid.org/0000-0002-4873-7488

Supplementary Data

Supplementary table S1: buffers and solutions

| **buffer/solutions** | **chemicals with final concentration** |
| --- | --- |
| isolation buffer with BSA (**IP+**); 250 mL; pH = 7.2 | - sucrose 0.3 M - TES 5 mM - EGTA 0.2 mM - BSA 0.1 % - KOH (for pH adjustment) |
| isolation buffer without BSA (**IP-**); 250 mL; pH = 7.2 | - sucrose 0.3 M - TES 5 mM - EGTA 0.2 mM - KOH (for pH adjustment) |
| 4x laemmli | - 1 M Tris/HCl pH 6.8 200 mM - sodium dodecyl sulfate 8 % - bromophenol blue 0.4 % - glycerol 40 % |
| 4x laemmli with ß-mercaptoethanol | - 4x Laemmli 500 µL - ß-mercaptoethanol 25 µL |
| 10x TBS pH 7.6 | - TRIS 200 mM - NaCl 1.37 M - HCl (for pH adjustment) |
| 0.5 M TRIS/HCl pH 6.8 | - TRIS 0.5 M - HCl (for pH adjustment) |
| 1 M TRIS/HCl pH 6.8 | - TRIS 1 M - HCl (for pH adjustment) |
| 1.5 M TRIS/HCl pH 8.8 | - TRIS 1.5 M - HCl (for pH adjustment) |
| 1x TBS | - 10x TBS 1x - H_2_O |
| TBS-T | - 10x TBS 1x - tween-20 0.1 % |
| 10x electrophoresis buffer | - TRIS 25 mM - SDS 1 % - H_2_O |
| 1x electrophoresis buffer | - 10x electrophoresis buffer 1x - glycin 192 mM - H_2_O |
| transferring buffer | - glycin 39 mM - TRIS 48 mM - SDS 0.025 % - EtOH 20 % - H_2_O |
| ponceau staining solution | - ponceau S 0.1 % - acetic acid 5 % - H_2_O |
| ponceau destaining solution | - acetic acid 10 % - EtOH 40 % - H_2_O |

Supplementary table S2: TE content of the EF Bio-Serv F1850 and V1534 diet

| **trace element** | **EF Bio-Serv F1850 (HFD)** | **V1534 (SD)** |
| --- | --- | --- |
| Fe [mg/kg] | 55 | 186 |
| Mn [mg/kg] | 25 | 68 |
| Zn [mg/kg] | 50 | 91 |
| Cu [mg/kg] | 12 | 15 |
| I [mg/kg] | 0.3 | 2.1 |
| Se [mg/kg] | 0.2 | 0.3 |

| **gel type** | **chemicals with final volume** |
| --- | --- |
| 4% stacking gel | - 0.5 M TRIS/HCl pH 6.8 25% - acrylamide 3.9% - SDS 0.1% - APS 0.05% - TEMED 0.1% - H_2_O |
| 12% running gel | - 1.5 M TRIS/HCl pH 8.8 25% - acrylamide 12% - SDS 0.1% - APS 0.05% - TEMED 0.05% - H_2_O |

Supplementary table S3: stacking and running gels

Supplementary table S4: primary antibodies, manufacturer, origin species and applied dilutions

| **abbreviation** | **protein** | **manufacturer** | **number** | **dilution** | **species** |
| --- | --- | --- | --- | --- | --- |
| VDAC | voltage-dependent anion channel | Cell Signaling Technology ® | 866 | 1:1000  (5% TBS-T) | rabbit |
| COX IV | cytochrome C oxidase Subunit 4I1 | Cell Signaling Technology ® | 4844 | 1:1000  (5% TBS-T) | rabbit |
| HSP 60 | heat shock protein 60 | BD Biosciences™ | 611562 | 1:5000  (5% TBS-T) | mouse |
| Cyt. C | cytochrome C | Cell Signaling Technology ® | 4272 | 1:1000  (5% TBS-T) | rabbit |
| Histone 3 | histone h3 | Cell Signaling Technology ® | 96495 | 1:1000  (5% TBS-T) | rabbit |
| BiP | binding immunoglobulin protein | BD Biosciences™ | 610978/9 | 1:250  (5% TBS-T) | mouse |
| LAMP2 | lysosomal associated membrane protein 2 | Invitrogen™ | 51-2200 | 1:2000  (5% TBS-T) | rabbit |
| ACT2 | aconitase 2 | Cell Signaling Technology ® | #6922 | 1:1000  (5% BSA) | rabbit |
| CS | citrate synthase | Proteintech Group, Inc. | #16131-1-AP | 1:1000  (5% TBS-T) | rabbit |
| β-actin | β-actin* | abcam | ab49900 | 1:2000  (5% TBS-T) | rabbit |

Supplementary table S5: ICP-MS/MS parameters

| **ICP-MS/MS parameter** | |
| --- | --- |
| plasma RF power | 1550 W |
| nebulizer | MicroMist (Glass Expansion, Melbourne Australia) |
| cones | Nickel |
| nebulizer gas flow | 0.98 L/min |
| makeup gas flow | 0.23 L/min |
| spray chamber | Scott type double pass spray chamber |
| spray chamber temperature | 2 °C |
|  |  |
| gas mode | He |
| collision / reaction gas flow | 3 mL/min |
| m/z Q1 and Q3 | 55 (Mn); 56 (Fe); 63 (Cu); 72 (Ge); 103 (Rh) |
| integration time | 0.5 s (Mn); 0.3 s (Fe; Cu, Ge; Rh) |
| replicates | 3 |
| sample depth | 8.0 mm |
| sweeps | 100 |
| tune maximum sensitivity on | 59→59 (Co); 89→89 (Y); 205→205 (Tl) |
