# Supplementary material for: Subzero project: comparing trace element profiles of enriched mitochondria fractions from frozen and fresh liver tissue
Source: Anal Bioanal Chem. 2024 Jul 3;416(20):4591–604. doi: 10.1007/s00216-024-05400-y (PMC11294433; doi:10.1007/s00216-024-05400-y)
Supplement: Supplementary file 2 — Supplementary file2 (DOCX 378 KB) [file 216_2024_5400_MOESM2_ESM.docx]

**Article Title:**

Sub Zero Project: Comparing trace element profiles of enriched mitochondria fractions from frozen and fresh liver tissue

**Jounral Name:**

Analytical and Bioanalytical Chemistry

**Author Names:**

Tom Heinze^1,2^, Franziska Ebert^1^, Christiane Ott^2,3^, Judith Nagel^4^, Carola Eberhagen^5^, Hans Zischka^4,5^, Tanja Schwerdtle^1,2,6, *^

^1^Institute of Nutritional Science, Department of Food Chemistry, University of Potsdam, Nuthetal, Germany ^2^TraceAge – DFG Research Unit on Interactions of Essential Trace Elements in Healthy and Diseased Elderly (FOR 2558), Berlin-Potsdam-Jena-Wuppertal, Germany
^3^Department of Molecular Toxicology, German Institute of Human Nutrition, Nuthetal, Germany
^4^Institute of Toxicology and Environmental Hygiene, Technical University Munich, School of Medicine and Health, München, Germany
^5^Institute of Molecular Toxicology and Pharmacology, Helmholtz Munich, München, Germany
^6^German Federal Institute for Risk Assessment (BfR), Berlin, Germany

*Corresponding author: [tanja.schwerdtle@uni-potsdam.de](mailto:tanja.schwerdtle@uni-potsdam.de)

orcid.org/0000-0002-4873-7488


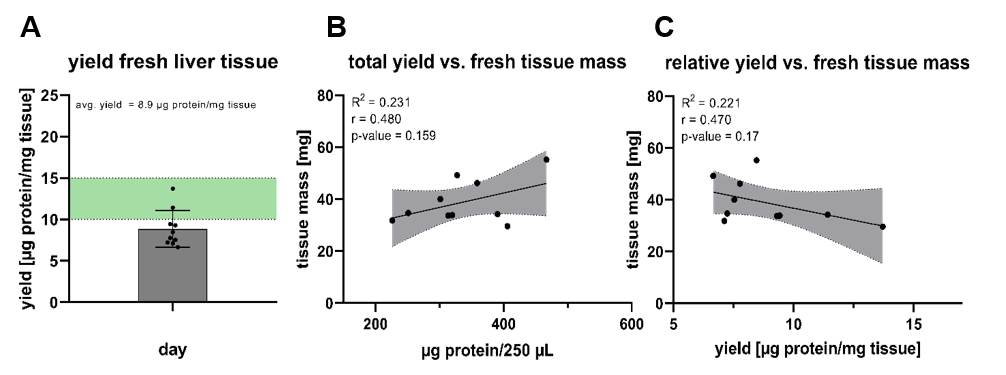


**Supplementary Figure 1** **Sufficient protein yield from frozen murine EMF correlates with total homogenized tissue mass**

Relative EMF protein yield in μg/mg fresh murine liver tissue homogenized, with the targeted yield range in green (A). Correlation between the absolute (B) or relative (C) EMF protein yield with the homogenized tissue mass. Pearson correlation and ordinary one-way ANOVA with Tukey's multiple comparisons test with * p ≤ 0.05; ** p ≤ 0.01 and *** p ≤ 0.001


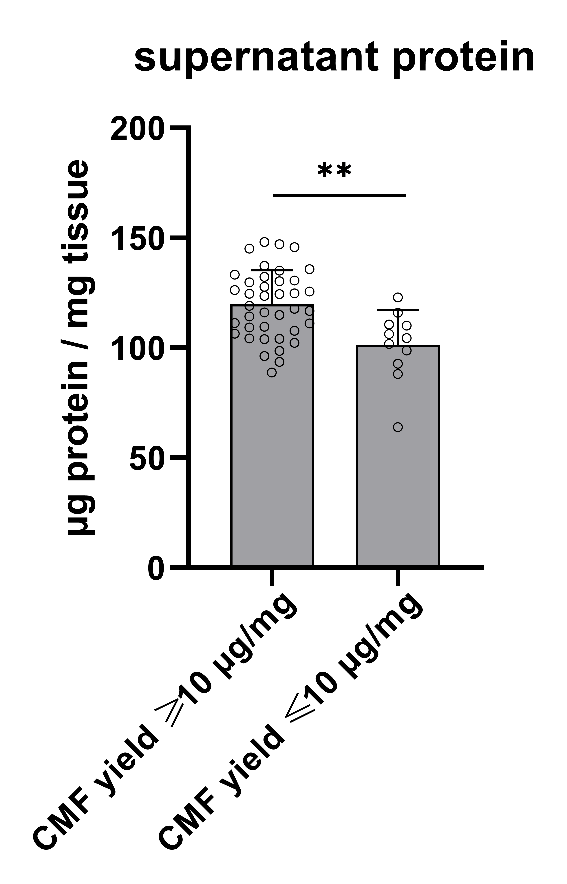


**Supplementary Figure 2 Protein content of the 9000 xg supernatant is indicative for tissue homogenization efficiency**

Protein content of the leftover 9000 xg supernatant of all EMFs from the mice that were used for method validation were Analyzed *via* Bradford assay. Samples were grouped based on EMF yield below or above 10 µg/mg tissue. Statistical analysis *via* unpaired, two-sided t test with * p ≤ 0.05; ** p ≤ 0.01 and *** p ≤ 0.001.

**
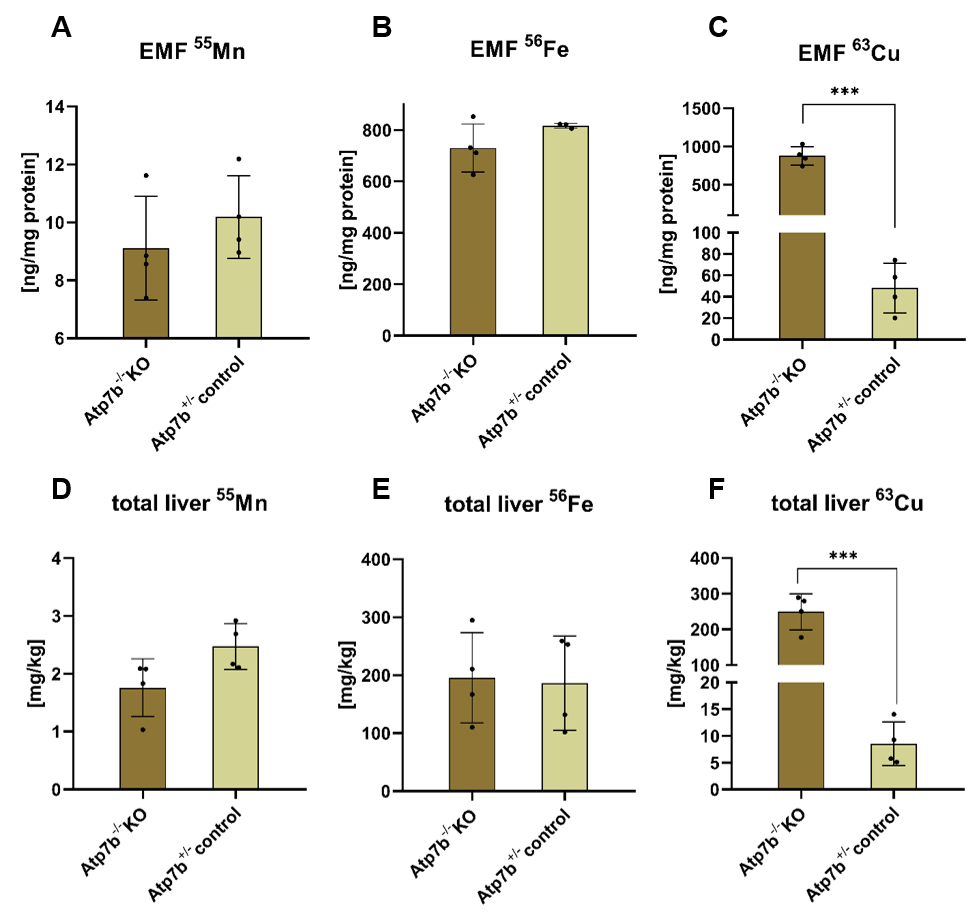
**

**Supplementary Figure 3 Mn, Fe and Cu content of EMFs and the corresponding total tissue of frozen rat liver of a Wilsons disease model**

Frozen tissue EMF Mn (A), Fe (B) and Cu (C) concentrations and total hepatic Mn (D), Fe (E) and Cu (F) concentrations per kg wet weight of homozygous *Atp7b-/-* knock out rats (dark brown) compared to heterozygous *Atp7b+/-* control rats (light brown). Statistical analysis *via* unpaired t-test with * p ≤ 0.05; ** p ≤ 0.01 and *** p ≤ 0.001.
